# Association between hemoglobin-to-red cell distribution width ratio and occurrence of sepsis during ICU stay in inflammatory bowel disease patients who died: a retrospective study using the EICU-CRD database

**DOI:** 10.1371/journal.pone.0353237

**Published:** 2026-07-07

**Authors:** Yulin Li, Qian Hua, Zhengyang Li, Miao Jiang

**Affiliations:** Department of Gastroenterology, Jinshan Hospital of Fudan University, Shanghai, China; The University of Lahore, PAKISTAN

## Abstract

**Background:**

The hemoglobin-to-red cell distribution width ratio (HRR) is a biomarker associated with systemic inflammation and outcomes in critical illness. Within clinical databases, there exists an extreme-prognosis subgroup of inflammatory bowel disease (IBD) patients, those who all died within 30 days of ICU admission. Due to limitations in the database, this study can only analyze this specific subgroup.

**Objective:**

This study aims to explore and describe the association between admission HRR and the occurrence of sepsis during ICU stay in this specific subgroup of IBD patients.

**Methods:**

A retrospective cohort study was conducted using the eICU Collaborative Research Database (2014–2015), including 229 eligible patients. Multivariable logistic regression was used to assess the independent association, adjusting for confounders. The dose-response relationship was examined using restricted cubic spline (RCS) models. Subgroup and interaction analyses were performed across age, sex, and race.

**Results:**

The sepsis group had a significantly lower admission HRR than the non-sepsis group (6.04 ± 1.66 vs. 6.77 ± 2.0, P = 0.008). After full adjustment, each 1-unit increase in HRR was associated with 20.3% lower odds of sepsis (P = 0.007). Compared to the lowest quartile (HRR < 5.1), patients in the highest quartile (HRR ≥ 7.85) had 66.8% lower adjusted odds of sepsis (P = 0.015). RCS analysis indicated a linear, inverse relationship between HRR and sepsis (P for non-linearity = 0.42). Subgroup analysis revealed the negative association was more pronounced in patients aged <65, males, and White patients. However, formal interaction tests were not statistically significant (all P for interaction >0.05), indicating the association did not differ meaningfully across these subgroups.

**Conclusions:**

In this specific cohort, a lower admission HRR was associated with the occurrence of sepsis. Due to inherent selection bias, this finding describes an association within this specific subgroup and cannot be generalized. This exploratory study generates the hypothesis that HRR may reflect a unique pathophysiological state in end-stage IBD, a hypothesis that requires validation in prospective, unbiased cohorts.

## Introduction

Inflammatory bowel disease (IBD) is a group of heterogeneous disorders marked by chronic gut inflammation [1]. IBD comprises three subtypes: Crohn’s disease (CD), ulcerative colitis (UC), and indeterminate colitis. Among these, CD and UC are the predominant forms, whereas indeterminate colitis is less common. In most adult clinical cohorts, IBD follows a decades-long, relapsing course. It brings a cascade of burdens ranging from chronic gastrointestinal symptoms, malnutrition, and immune dysfunction to a suite of extraintestinal complications, all of which erode patients’ day-to-day quality of life and cut long-term life expectancy short [2]. About 1.4% patients in IBD will require admission to ICU as their condition advances, most often triggered by uncontrolled acute flares or severe, life-threatening complications [3]. This specific patient cohort faces greater therapeutic challenges and poorer prognoses, especially if sepsis becomes a complication. If sepsis is a complication, the mortality rate can reach 34.9% [4,5], much higher than the general population with sepsis [6]. Therefore, identifying biomarkers associated with adverse outcomes in critically ill IBD patients is of significant interest.

In recent years, derivative markers from routine blood tests have been used to predict outcomes of many diseases, owing to their simplicity, low cost, and good reproducibility [7–10], such as the neutrophil-to-lymphocyte ratio (NLR) [7], platelet count [8], the RDW-to-platelet ratio (RPR) [9], red cell distribution width (RDW) [10], and the ratio of neutrophil counts to prognostic nutritional index [11]. RDW, in particular, has been proved to be associated with poor outcomes in several chronic inflammations and sepsis [12–14]. Hemoglobin (Hb), which is a basic measure of oxygen-carrying ability and nutritional status, is often low in IBD patients due to disease activity, malnutrition, and ongoing blood loss [15,16]. Hemoglobin-to-Red Cell Distribution Width Ratio (HRR) is a newer composite marker combining information from Hb and RDW. It has recently shown promise for predicting outcomes in heart disease, cancers, and sepsis [17–22]. HRR provides information about two physiological aspects: 1) falling Hb indicates impaired oxygen delivery and disease activity; 2) high RDW indicates inflammation-driven red blood cell production and increased oxidative stress [23]. In theory, a lower HRR could mean more severe systemic inflammation, poorer nutrition, and more tissue hypoxia-causing sepsis to develop and worse.

However, existing studies have primarily focused on the prognostic value of HRR in patient cohorts that include both survivors and non-survivors. Within clinical databases, there is a subgroup of IBD patients who experience extremely poor outcomes, specifically those who all die within 30 days after being admitted to ICU. The clinical course and complication patterns in this particular subgroup may be unique, yet a thorough analysis of the associations between clinical characteristics, such as biomarkers, and complications like sepsis within this exclusively non-surviving subgroup remains unexplored. Understanding biomarker patterns in this subgroup, although with limited clinical generalizability, holds exploratory value for completing the portrait of the critical IBD disease spectrum.

Consequently, this study is an exploratory, descriptive analysis. Using data from the eICU Collaborative Research Database (eICU-CRD) [24], we have specifically limited our study population to IBD patients who died within 30 days of ICU admission. The primary aim of this study is to explore the association between admission HRR and the occurrence of sepsis during the ICU stay within this specific subgroup of non-survivors with extreme outcomes. We acknowledge that, due to the pre-selected population based on mortality outcomes, this study contains a significant selection bias. Our findings are meant only to interpret associations within this subgroup and are not applicable to surviving or general critically ill IBD patients.

## Methods

### Data source

This study is a retrospective, observational analysis based on the eICU-CRD v2.0 database. This database was jointly developed by Philips Healthcare and the MIT Laboratory for Computational Physiology, consisting of more than 200,000 ICU patients in 208 US hospitals, collected in 2014–2015, including ICU, e.g., hourly vital signs, laboratory tests, medication records, and APACHE scores. Data were used according to the database usage guidelines and registered through official registration.

### Study population

Due to database constraints and the specific analytical objectives of this study, we screened all adult patients (aged ≥18 years) admitted to the ICU between 2014 and 2015. The inclusion criteria were: (1) age ≥ 18 years; (2) diagnosis of IBD in terms of ICD-9-CM codes for all subtypes (except Crohn’s disease code 555.* and ulcerative colitis code 556.*). For diagnostic specificity, 555.9 (Crohn’s disease unspecified) and 556.9 (ulcerative colitis unspecified) were prioritized first, and then extended to all subtype codes 555.*,556.* to maximize the number of eligible patients; (3) complete hemoglobin (Hb) and red blood cell distribution width (RDW) measurements within 24 hours after ICU admission for HRR calculation.

The exclusion criteria included: (1) missing HRR data or incomplete baseline information. Sepsis was diagnosed in accordance with the Sepsis-3 international consensus criteria [25]: (1) presence of suspected or confirmed infection; (2) an increase in the Sequential Organ Failure Assessment (SOFA) score of ≥ 2 points from baseline.

Because the database prioritizes acute ICU conditions, detailed subtype classification of chronic diseases like IBD may not be available. Some patients may only be identified with “unspecified” subtype codes (555.9, 556.9), even after all subtype codes, some IBD patients may not be accounted for because of limited codes. This limitation is inherent to the database. We acknowledge it.

### Outcome measure

The primary outcome was the occurrence of sepsis during the ICU stay among patients who died within 30 days of ICU admission.

### Exposure and covariates

The primary exposure was hemoglobin/RDW (HRR), calculated from the first Hb and RDW values measured within 24 hours of ICU admission. HRR was analyzed both as a continuous variable and a categorical variable (quartiles: Q1 < 5.1, Q2: 5.1–6.32, Q3: 6.33–7.85, Q4 ≥ 7.85).

Covariates included demographic, clinical, and laboratory variables: age, sex (male/female), race (African American, White, or other), body weight, serum calcium levels, red blood cell (RBC) count, body temperature, APACHE IV score, APS score, delirium score, GCS score, OASIS score, and SOFA score.

### Data extraction and quality control

Data were extracted from eICU-CRD v2.0 using R software (version 4.4.3). Extracted variables included demographics, ICD-9-CM diagnosis codes, laboratory results (Hb, RDW, calcium, platelet count, RBC count, WBC count), vital signs (temperature), severity scores (APACHE, APS, delirium, GCS, OASIS, SOFA), and outcome data. Quality control measures included consistency checks of data fields and the identification of outliers. Samples with missing key variables were excluded.

### Statistical analysis

All analyses were performed using R 4.4.3. Continuous variables were described as mean ± standard deviation (SD) or median (interquartile range, IQR) based on distribution. Categorical variables were presented as counts (percentages). Group comparisons were conducted using the Wilcoxon rank-sum test (continuous variables) and Rao-Scott chi-squared test (categorical variables).

Given the inherent selection bias of our study population (all patients deceased), the statistical analyses were performed with an exploratory and descriptive purpose. We used multivariate logistic regression models to assess the association between HRR and sepsis. HRR was considered both as a continuous variable and a categorical variable (quartiles, Q1–4). Three models were used: 1 (unadjusted), 2 (adjusted for age, sex, and race), 3 (adjusted for body weight, calcium, RBC count, temperature, and SOFA score as continuous covariates). Odds ratios (ORs) and 95% CIs were reported. Restricted cubic splines (RCS) were used for the dose-response relationship and test for non-linearity. Subgroups were analysed by age (65 vs. 65 years), gender (male vs. female), and race (African American, White, or other). Interaction terms (HRR subgroup factor) were tested. All tests were two-tailed, and statistical significance was P 0.05. P-values for multiple comparisons were corrected using the false discovery rate (FDR).

### Multicollinearity assessment

To ensure the robustness of the regression estimates, we assessed multicollinearity among all covariates included in the final model using the variance inflation factor (VIF). A VIF value < 10 was considered indicative of no severe multicollinearity.

## Results

### Baseline results

**Fig 1:**
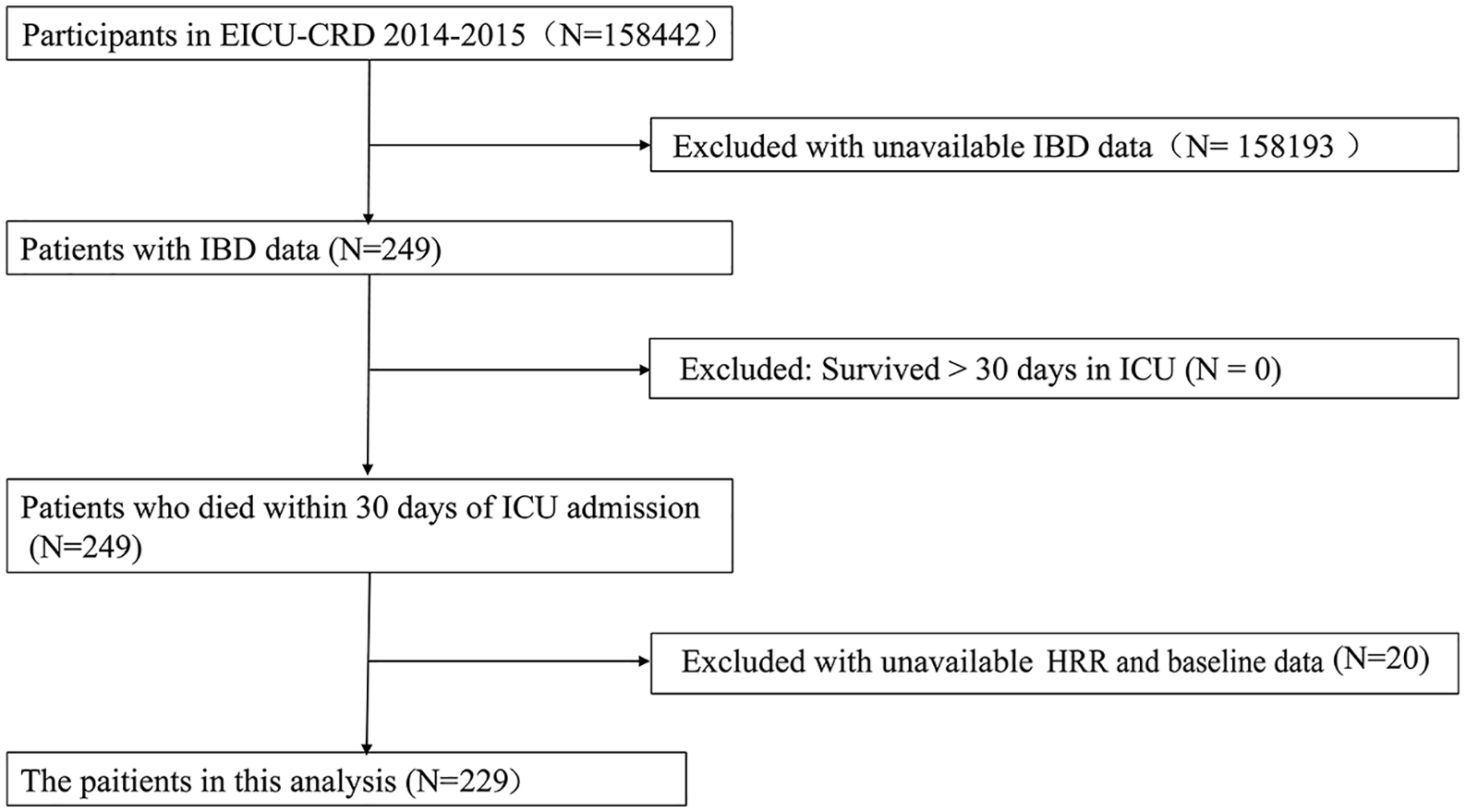
Flowchart of participant selection. IBD, Inflammatory Bowel Disease.

A total of 229 IBD patients who died within 30 days of ICU admission were enrolled in the final analysis. The selection process is shown in (Fig 1). From the initial 158,442 ICU admissions in the eICU-CRD (2014–2015), 249 patients were diagnosed with IBD according to ICD-9-CM codes (555.* for Crohn’s disease and 556.* for ulcerative colitis). Since this study focused on terminal outcome, we selected those who died within 30 days of ICU admission (No patients survived beyond this period, so we excluded 20 patients because of missing HRR or other baseline data), 229 patients were selected. Among them, 74 patients developed sepsis during the ICU stay (Sepsis Group) and 155 patients did not (Non-sepsis Group). Baseline characteristics of the two groups are summarized in Table 1. Significant differences were observed between the Sepsis and Non-sepsis groups in terms of body weight (P = 0.019), serum calcium (P = 0.001), hemoglobin (P = 0.003), red blood cell count (P < 0.001), SOFA score (P = 0.011), body temperature (P = 0.006), and HRR (P = 0.008). There are no significant differences for age, sex, height, race, creatinine, platelet count, RDW, white blood cell count, APACHE score, APS score, Delirium score, GCS score, or OASIS score (all P > 0.05).

**Table 1:** Patient characteristics.

Variables	Overall	Sepsis	Non-Sepsis	p-value^2^
	N = 229^1^	N = 74^*1*^	N = 155^*1*^	
**Age**	55 (18)	56 (18)	54 (19)	0.51
**Age (%)**				0.59
Young (<65 y)	148 (65%)	46 (62%)	102 (66%)	
Old (≥65 y)	81 (35%)	28 (38%)	53 (34%)	
**gender**				0.22
Female	126 (55%)	45 (61%)	81 (52%)	
Male	103 (45%)	29 (39%)	74 (48%)	
**height**	166 (21)	164 (25)	168 (18)	0.18
**race**				0.43
African American	27 (12%)	6 (8.1%)	21 (14%)	
Caucasian	181 (79%)	62 (84%)	119 (77%)	
Other	21 (9.2%)	6 (8.1%)	15 (9.7%)	
**Weight (kg)**	78 (23)	83 (24)	75 (22)	0.019
**Calcium (mg/dL)**	7.99 (0.82)	7.79 (0.75)	8.08 (0.84)	0.001
**creatinine (mg/dL)**	1.63 (2.49)	1.40 (1.68)	1.73 (2.79)	0.84
**HGB (g/dL)**	10.13 (2.21)	9.56 (2.03)	10.41 (2.25)	0.003
**platelet count (1,000 cells/uL)**	242 (123)	252 (141)	238 (114)	0.61
**RBC (1,000 cells/uL)**	3.51 (0.75)	3.29 (0.63)	3.61 (0.78)	<0.001
**RDW (%)**	16.01 (2.55)	16.28 (2.63)	15.88 (2.51)	0.17
**WBC (1,000 cells/uL)**	12.5 (7.4)	13.7 (8.5)	11.9 (6.8)	0.064
**Temperature(°C)**	36.90 (0.77)	37.16 (0.92)	36.77 (0.66)	0.006
**Apache_score**	53 (22)	56 (23)	51 (22)	0.18
**Aps_score**	44 (20)	47 (20)	42 (20)	0.11
**Delirium_score**	18 (7.9%)	7 (9.5%)	11 (7.1%)	0.53
**Gcs_score**	14.19 (2.01)	14.11 (2.30)	14.23 (1.86)	0.75
**Oasis_score**	24 (8)	26 (8)	24 (8)	0.052
**Sofa_score**	3.24 (2.83)	3.89 (3.06)	2.92 (2.66)	0.011
**HRR**	6.53 (1.92)	6.04 (1.66)	6.77 (2.00)	0.008

^1^Mean (sd) or Frequency (%)

^2^Wilcoxon rank sum test; Pearson’s Chi-squared test

HGB: Hemoglobin, RBC count: Red Blood Cell count, RDW: Red Cell Distribution Width, WBC count: White Blood Cell count, HRR: Hemoglobin-to-red blood cell distribution width ratio

### Association between HRR and sepsis occurrence

Table 2 presents the results of the multivariable logistic regression analysis examining the association between HRR (as a continuous and quartile-categorized variable) and sepsis occurrence. Analyzed as a continuous variable, HRR showed a significant inverse association with sepsis in the unadjusted model (Model 1: OR: 0.808, 95% CI: 0.687–0.941; P = 0.008). This inverse association remained significant after adjusting for demographic factors (Model 2: OR: 0.786, 95% CI: 0.664–0.921; P = 0.004) and after full adjustment for confounders (Model 3: OR: 0.797, 95% CI: 0.672–0.937; P = 0.007). Specifically, each unit increase in HRR was associated with an approximate 20.3% reduction in sepsis occurrence. When HRR was categorized into quartiles with Q1 as the reference, a significant dose-response trend was observed (P for trend = 0.007). In the fully adjusted Model 3, patients in the highest HRR quartile (Q4) had a 66.8% lower odds of sepsis compared to those in Q1 (OR = 0.332, 95% CI: 0.131–0.793; P = 0.015). The protective effects for Q2 and Q3 were not statistically significant (both P > 0.05), suggesting a potential threshold effect. Assessment of multicollinearity in the final model (Model 3) using variance inflation factors (VIF) confirmed that all variables had VIF values below 10 (Table 3), indicating no severe multicollinearity.

**Table 2:** The relationship between HRR and sepsis.

	Model 1			Model 2			Model 3		
Characteristic	OR	95% CI	P-value	OR	95% CI	P-value	OR	95%CI	P-value
**HRR**									
**HRR continuous**	0.808	(0.687,0.941)	0.0075	0.796	(0.671,0.934)	0.0066	0.779	(0.649,0.927)	0.0058
**HRR quantile**									
**Q1 (low)**	Ref	Ref		Ref	Ref		Ref	Ref	
**Q2**	1.157	(0.547,2.457)	0.7027	1.131	(0.528,2.428)	0.7506	1.26	(0.558,2.861)	0.5777
**Q3**	0.734	(0.337,1.586)	0.433	0.662	(0.298,1.455)	0.3067	0.702	(0.297,1.637)	0.4138
**Q4 (high)**	0.331	(0.135,0.771)	0.0124	0.328	(0.131,0.779)	0.0137	0.279	(0.101,0.724)	0.0106
**p for trend**			0.0074			0.0072			0.0067

OR, odds ratio; CI, confidence intervals; HRR, hemoglobin-to-red blood cell distribution width ratio.

HRR: Q1:HRR< 5.1; Q2: 5.1≤ HRR<6.34; Q3: 6.34≤HRR<7.85; Q4: HRR≥ 7.85.

The model 1 was the crude model.

The model 2 was adjusted by Age, Gender, Race.

The model 3 was adjusted by Age, Gender, Race, Weight, Calcium, WBC count, Temperature.

**Table 3:** Variance inflation factor (VIF) values for predictors in Model 2 and Model 3.

Variable	Model2	Model3
**Age**	1.01644802436201	2.41627541753341
**Gender**	1.16232471500129	1.24540001643584
**Height**	1.20773537842054	1.37213206897895
**Race**	1.03193722094987	1.3692394366239
**Weight**	1.06700893510819	1.18368895696444
**Calcium**		1.20056282801289
**Creatinine**		1.38148581111679
**HGB**		5.82691505454699
**Platelet count**		1.93424367817006
**RBC**		5.41351822609371
**RDW**		1.61248584830909
**WBC**		1.24474094715154
**Temperature**		1.14013430239362
**Apache_score**		8.55233116630879
**Aps_score**		7.50006609012461
**Delirium_score**		1.26878089336086
**Gcs_score**		2.50356756875535
**Oasis_score**		1.91581145827874
**Sofa_score**		2.74899328870677
**HRR**		2.01172270814008

HGB: Hemoglobin, RBC count: Red Blood Cell count, RDW: Red Cell Distribution Width, WBC count: White Blood Cell count, HRR: Hemoglobin-to-red blood cell distribution width ratio

### Dose-response relationship between HRR and sepsis

**Fig 2:**
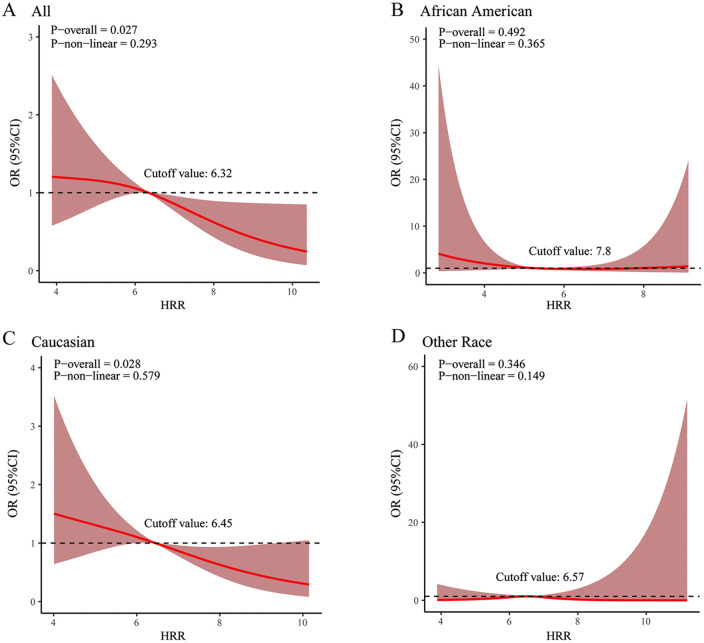
RCS Curves of the association between HRR and sepsis. (A: Total, B: African American, C: Caucasian, D: Other Race).

To further characterize the shape of the association between HRR and sepsis occurrence, we employed restricted cubic spline (RCS) models (Fig 2A). After multivariable adjustment, a statistically significant overall association was found (P for overall association = 0.027), and the relationship was linear (P for nonlinearity = 0.293). The risk curve showed that when HRR reached 6.32, the upper bound of the 95% CI for the odds ratio (OR) always fell below 1, suggesting that higher HRR was associated with a significantly reduced odds of sepsis.

RCS analyses stratified by race found a significant linear inverse association among White patients (P for overall association = 0.028; P for nonlinearity = 0.579) (Fig 2C), with a significantly lower probability for sepsis at HRR of approx. 6.45. However, no significant association was detected in the African American (Fig 2B) or “Other” race (Fig 2D) subgroups (both P for overall > 0.3). Due to limited sample sizes, these results need cautious interpretation.

### Subgroup analyses

**Fig 3:**
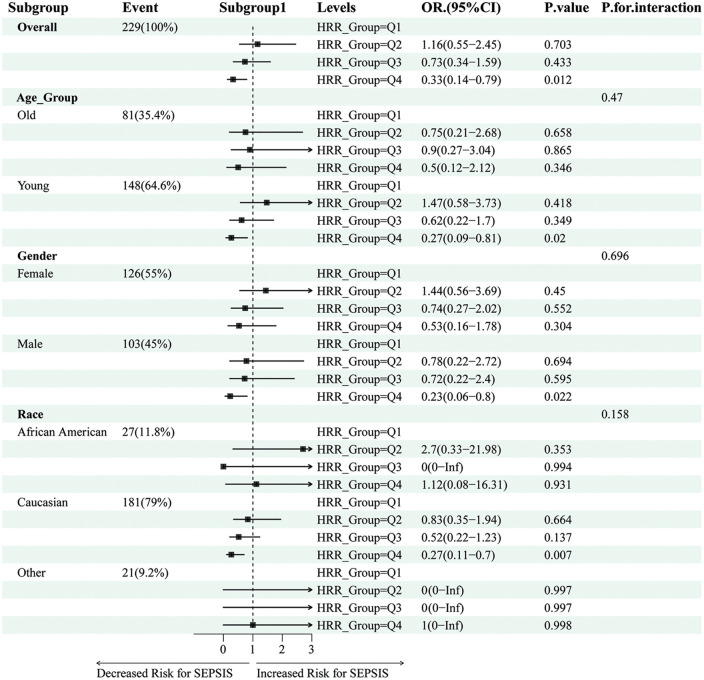
Subgroup and interaction analyses across age, sex, and race.

Subgroup analyses were performed to investigate potential heterogeneity (Fig 3). Within this certain subgroups, a high HRR (Q4) was statistically linked to reduced sepsis odds in patients aged <65 years (OR = 0.27, 95% CI: 0.09–0.81; P = 0.02), males (OR = 0.23, 95% CI: 0.06–0.80; P = 0.022), and White patients (OR = 0.27, 95% CI: 0.11–0.70; P = 0.007). But interaction tests showed no statistically significant differences across age (<65 vs. ≥ 65 years), sex (male vs. female), or race subgroups (all P for interaction > 0.05), suggesting that the association was consistent across these subgroups.

## Discussion

This study focused on a strictly defined subgroup with extreme outcomes due to data availability, IBD patients who died within 30 days of ICU admission. We conducted a descriptive retrospective analysis to examine the association between the admission hemoglobin-to-red cell distribution width ratio (HRR) and the incidence of sepsis during the ICU stay in this specific cohort. We found that a lower HRR was independently associated with a higher likelihood of sepsis in this cohort. The relationship was linear and inverse, with a significant reduction in sepsis odds for higher HRR values and a marked difference between the highest and lowest HRR quartiles. Subgroup analyses suggested the association may be more pronounced in males, patients aged <65, and White individuals, although interaction tests were not statistically significant.

The correlation between HRR and adverse outcome is consistent with its new role as a predictor of mortality in many critical diseases. For instance, a large population-based study using data from the NHANES database showed that patients with higher HRR had lower risk of cardiovascular disease and all-cause mortality [26]. Similarly, in patients with sepsis, chronic obstructive pulmonary disease, and coronary heart disease, lower HRR had higher risk of long-term mortality [21,27–29]. This pattern suggests that HRR captures a core pathophysiological state (low hemoglobin) and high inflammation and oxidative stress (high RDW) in patients with a high risk of complications [26,30]. On the surface, our findings align directionally with existing literature. However, it is essential to highlight the fundamental differences between this study and previous research. Most previous prognostic studies of HRR, including those cited before, assess its ability to predict future events in mixed cohorts of survivors and non-survivors [26–30]. In contrast, our study concentrated on a pure mortality cohort, investigating HRR’s association with an imminent, terminal complication. This is consistent with increasing evidence supporting the prognostic value of ratio-based biomarkers in sepsis. The serum glucose-potassium ratio has been linked to short- and long-term all-cause mortality in ICU patients with sepsis [31]. The creatinine-to-albumin ratio has shown a non-linear association with medium-term mortality in sepsis with acute kidney injury [32]. Additionally, the neutrophil-to-prognostic nutritional index ratio shows a J-shaped association with mortality [11]. Collectively, these findings highlight composite biomarkers from routine tests for risk stratification. Therefore, the objective here was not to “predict” sepsis but to explore HRR’s connection with a proximate, terminal complication within this mortality cohort.

Given the fundamental limitation outlined, we cautiously attempt a pathophysiological interpretation of the observed association within this non-survivor subgroup. A low HRR may indicate a severe state of biological dysregulation. First, low HRR reflects anemia of chronic inflammation and impaired erythropoiesis [22]. Patients with IBD often have persistent intestinal inflammation that leads to anemia. Causes include intestinal bleeding, iron deficiency, anemia of inflammation, and side effects of drugs [16]. At the same time, elevated RDW indicates anisocytosis [33]. Increased RDW is associated not only with anemia but also systemic inflammation and oxidative stress [13]. Inflammation and oxidative stress can disrupt red blood cell maturation, resulting in a shorter erythrocyte lifespan and variable size [34]. Thus, low HRR can serve as an indicator of anemia severity and the activity of inflammation/oxidative stress [35]. Second, low HRR may increase sepsis risk in IBD patients due to impaired intestinal barrier function and bacterial translocation [36]. Active intestinal inflammation compromises the gut barrier, while anemia and malnutrition can impair mucosal repair and immune defense. The inflammatory and oxidative stress environment reflected by low HRR further exacerbates intestinal injury, increasing the risk of bacteria and endotoxins entering the systemic circulation and thereby raising the likelihood of sepsis. Chronic inflammatory anemia and oxidative stress can lead to immune cell dysfunction, affecting the metabolism and bactericidal capacity of immune cells and reducing the ability to clear pathogens. Low HRR marks a high inflammatory load, which may lead patients to uncontrolled systemic inflammation and rapid progression to sepsis. Additionally, patients with sepsis often have low serum calcium levels, which supports the association between metabolic disturbances and sepsis risk. Calcium ions play a key role in immune cell signaling and inflammatory responses, and hypocalcemia is a marker of severity in critical illness and sepsis [37]. Low HRR in critically ill IBD patients may indicate a vicious cycle of chronic inflammation, oxidative stress, anemia, and dysregulation of immune metabolism. This state damages the mucosal barrier, impairs immune surveillance, and increases infection risk, explaining its association with sepsis. However, this study lacks key clinical details (such as disease activity and use of drugs), so we cannot quantify the specific contributions of these factors, which is a limitation of the study.

Subgroup analyses suggested possible differences in the HRR-sepsis association. Point estimates indicated a stronger protective effect of high HRR in males, patients aged <65 years, and White patients, although formal interaction tests were not significant. The trend toward a stronger effect in younger patients is similar to a study on depression [38], which reported that the inverse association between HRR and depressive symptoms was more pronounced in adults aged ≥45 years, hinting at possible age-related effect modification. The apparent racial difference, with a significant association observed only in White patients, must be interpreted with great caution because of small subgroup numbers. This is consistent with known ethnic differences in RDW ranges and may reflect unmeasured genetic or socioeconomic factors influencing both IBD and sepsis risk. These preliminary signals need larger, more diverse cohorts to verify.

Our conclusions have several limitations. The most significant is inherent selection bias. As we focused only on deceased patients, we lack insights into the prognostic value of HRR for sepsis among ICU-admitted IBD survivors. Therefore, the identified HRR threshold (e.g., 6.32) applies only to similar end-stage populations and cannot be generalized to all ICU-admitted IBD patients. Other limitations include the retrospective study design and the use of administrative data, which leave residual confounding. In addition, key clinical details such as specific IBD phenotypes, disease activity indices, detailed medication histories, and primary infection sites were unavailable. Only a single baseline HRR measurement was used, while tracking its dynamic changes during the ICU stay could yield further insights. The utility of HRR as a risk stratification tool for sepsis in critically ill IBD patients must be further validated in large prospective cohorts including both survivors and non-survivors. Future research should aim to: (i) validate the predictive ability of HRR for sepsis in prospective cohorts of critically ill IBD patients with heterogeneous prognoses; (ii) monitor changes in HRR during the ICU stay, which may provide more valuable insights than single-timepoint measurements; (iii) combine HRR with existing clinical scores to optimize risk stratification and guide individualized management of these patients.

Despite these limitations, our study holds exploratory value. It is the first to describe the link between admission HRR and sepsis in a non-surviving subgroup of critically ill IBD patients, offering a unique perspective on this extreme-outcome cohort. The findings support HRR’s potential role as a composite index integrating anemia (hemoglobin) and inflammation/stress (RDW) to reflect physiological dysregulation in critically ill patients. Although non-generalizable, the association provides preliminary clues for systematic validation of HRR’s predictive value in prospective ICU-admitted IBD patient cohorts, including survivors.

## Conclusion

In this study, we focused on a subgroup with an extremely poor prognosis—all IBD patients who died within 30 days of ICU admission. Our analysis found that a lower HRR at admission was independently associated with an increased risk of sepsis during the ICU stay in this subgroup. However, due to data limitations, there is significant selection bias in this study. As all subjects were predetermined to be deceased patients, the findings can only describe associations within this subgroup and cannot be generalized to all IBD patients in ICU. The potential value of HRR as a risk stratification tool for sepsis must be validated in larger, prospective ICU-IBD patient cohorts including both survivors and non-survivors.

## Abbreviations

HRR: Hemoglobin to red cell distribution width ratio
IBD: Inflammatory Bowel Disease
CD: Crohn’s disease
UC: Ulcerative colitis
ICU: Intensive Care Unit
RCS: Restricted Cubic Spline
RDW: Red cell distribution width
EICU-CRD: eICU Collaborative Research Database
