# Supplementary material for: Association between hemoglobin-to-red cell distribution width ratio and occurrence of sepsis during ICU stay in inflammatory bowel disease patients who died: a retrospective study using the EICU-CRD database
Source: PLoS One. 2026 Jul 7;21(7):e0353237. doi: 10.1371/journal.pone.0353237 (PMC13340837; doi:10.1371/journal.pone.0353237)
Supplement: Attachment — Submitted filename: Response to Reviewers.docx [file pone.0353237.s002.docx]

Dear Editor,

Thank you for your email and the opportunity to revise our manuscript. We have carefully addressed all the points raised by the reviewers in the previous round and ensured that the manuscript now fully complies with PLOS ONE’s style and formatting requirements as outlined in your email. Below, we provide a detailed, point-by-point response.

In addition to the academic revisions, we would like to respectfully bring one administrative circumstance to your attention. The first author of this study is currently completing standardized specialist training in Shanghai, with a graduation requirement of having a manuscript published or officially accepted by May 30, 2026. This timeline is crucial for their professional certification. We understand that the review process requires thoroughness and scientific rigor. However, given this pressing deadline, we would be most grateful if you and the reviewers could consider providing your decision and feedback as expeditiously as possible, to allow us the opportunity to meet this essential career requirement.

We assure you that this personal circumstance in no way diminishes our commitment to the highest standards of scientific integrity and transparency, which we have strived to uphold in this revised manuscript. We sincerely hope that our thorough revisions and this explanation will merit your positive consideration.

1. **Style Requirements:** We have thoroughly reviewed and reformatted our manuscript to adhere to PLOS ONE’s style templates. We confirm that the manuscript now meets all formatting requirements regarding file naming, document structure, and reference style.

2. **Code Sharing:** We acknowledge PLOS ONE’s policy on code sharing. Our study is a retrospective analysis based on the eICU-CRD database, and the primary statistical analyses were performed using R 4.4.3. The analysis does not rely on novel, author-generated computational code or algorithms that underpin the findings. The statistical methods employed (e.g., multivariate logistic regression, restricted cubic splines) are standard and implemented using common R packages.

3. **Financial Disclosure:** As stated in the Funding section of our manuscript, “This work was supported by Reserve discipline construction of Jinshan Hospital Affiliated with Fudan University (No. ZDXK-2025-7)” 21. We confirm that the funders had no role in study design, data collection and analysis, decision to publish, or preparation of the manuscript. Please amend the online submission form accordingly.

4. & 5. **Figure References and Captions:** We have reviewed the entire manuscript and confirm that all figures (including Figure 2) are appropriately referenced in the text. Each figure is now accompanied by a separate, detailed caption.

6. **Figure 4:** We apologize for the oversight. Reference to “Figure 4” on page 12 was an error from a previous draft. We have removed all mention of Figure 4 from the manuscript text. The current submission contains only the figures that are essential and referenced.

7. **Reviewer-Recommended Citations:** We have reviewed the publications suggested by the reviewers in their comments. We have evaluated their relevance to our study’s context—specifically, the association between HRR and outcomes in critical illness and sepsis. We have accordingly cited several key and relevant publications that support the biological rationale and clinical context of our findings in the revised Discussion section.

Sincerely,

The Authors

Dear Reviewer,

We are deeply grateful for your insightful and constructive comments, which have significantly improved the quality and clarity of our manuscript. We have carefully considered each point and made substantial revisions accordingly. Below, we provide a detailed, point-by-point response.

Reviewer # 1:

Comment 1: “This study compared the relationship between sepsis and HRR in IBD patients who died in the ICU. The summary should emphasize that this is an analysis of the deceased population. Since the study does not involve predictive modeling, conclusions must be more rigorously stated.”

**Response:**

We sincerely thank the reviewer for this crucial point.

We have thoroughly revised the manuscript to consistently emphasize the exclusive focus on the deceased patient subgroup and to ensure conclusions are descriptive and non-predictive.

In the Abstract, we now explicitly state the study population as “IBD patients who all died within 30 days of ICU admission” and describe it as an “extreme-prognosis subgroup”. The objective is framed as exploring “the association between admission HRR and the occurrence of sepsis during ICU stay in this specific subgroup”.

Throughout the Results and Discussion, we consistently refer to “this subgroup” and describe findings as “associations within this subgroup”.

The Conclusion has been strengthened to state with precision: “As all subjects were predetermined to be deceased patients, the findings can only describe associations within this subgroup and cannot be generalized to all IBD patients in ICU” . We have removed any language that could be misconstrued as predictive.

Comment 2: “The objectives and importance of the study are inadequate. Why only deceased patients selected? There is a lack of theoretical basis and discussion about the mechanism of death in this disease.”

**Response:**

We appreciate this request for greater clarity on the study’s rationale and mechanistic context.

Study Rationale: We have expanded the Introduction to better explain the selection of this unique cohort. We clarify that due to the specific constraints of the eICU-CRD database and our analytical focus, the study is inherently limited to analyzing IBD patients with the extreme outcome of death within 30 days 0. We acknowledge this introduces significant selection bias but argue that characterizing this “extreme-prognosis subgroup” has exploratory value for understanding the full spectrum of critical IBD .

Pathophysiological Discussion: In the Discussion, we have enriched the interpretation of our findings. We elaborate on how a low HRR in this non-survivor cohort likely reflects a compounded state of severe inflammation, anemia of chronic disease, and potential nutritional impairment, which may contribute to vulnerability to sepsis through mechanisms like gut barrier dysfunction and immune dysregulation, as suggested by related literature on systemic inflammation 11. This provides a stronger theoretical basis for the observed association in this critically ill population.

Comment 3: “In the methods part, ‘survival more than 30 days after ICU admission’ as an exclusion criterion may introduce selection bias.”

**Response:**

The reviewer is absolutely correct. This is not merely a potential bias but the defining characteristic and primary limitation of our study design. We have made this transparent and central to the manuscript.

We have reframed the Methods (Study Population) section to clearly state that the study population consists specifically of “IBD patients who died within 30 days of ICU admission” .

This selection bias is now the lead point in the Limitations section: “The most significant [limitation] is inherent selection bias. As we focused only on deceased patients, we lack insights into the prognostic value of HRR for sepsis among ICU-admitted IBD survivors” .

Comment 4: “The sample size in statistical analysis was small, and there was a high risk of overfitting. Multicollinearity is not reported, and red blood cell counts are likely collinear with HRR.”

**Response:** We thank the reviewer for these important methodological points.

Sample Size and Overfitting: We acknowledge the limited sample size. To mitigate overfitting in our multivariate models, we employed a parsimonious strategy, adjusting only for a limited set of key covariates (e.g., age, gender, race, weight, calcium, WBC, temperature) based on clinical relevance and univariate analysis 711. We have added a cautionary note in the Limitations section regarding this .

Multicollinearity: Following the reviewer’s suggestion, we formally assessed multicollinearity for the final adjusted model (Model 3). We calculated the Variance Inflation Factor (VIF) for all included variables. As shown in the supplementary material (Table S1), all VIF values were well below 10 (e.g., RBC count VIF = 5.41, HRR VIF = 2.01), indicating that severe multicollinearity was not present 13. We have added a statement in the Statistical Analysis section noting that multicollinearity was assessed and was not a concern.

Comment 5: “The conclusion lacks rigor and contains overgeneralizations, and needs to be revised. The limitation should first address the selection bias that arises from the inclusion of only deceased patients.”

**Response:** We agree entirely and have comprehensively revised the conclusion and limitations to ensure rigor and specificity.

Primary Limitation: As noted in response to Comment, the Limitations section now begins by directly addressing the selection bias .

Revised Conclusion: The Conclusion has been rewritten to be strictly accurate and limited: “Our analysis found that a lower HRR at admission was independently associated with an increased risk of sepsis during the ICU stay in this subgroup of deceased IBD patients. However, due to data limitations, there is significant selection bias in this study. As all subjects were predetermined to be deceased patients, the findings can only describe associations within this subgroup and cannot be generalized to all IBD patients in ICU” .

We believe these revisions have significantly strengthened the manuscript by enhancing its transparency, methodological rigor, and by precisely framing the scope and implications of the findings. We are grateful for the reviewer’s valuable input.

We sincerely thank you again for your time and constructive guidance, which has greatly improved the quality of our manuscript. We look forward to your further feedback.

Sincerely,

The Authors
