# Supplementary material for: Association between hemoglobin-to-red cell distribution width ratio and occurrence of sepsis during ICU stay in inflammatory bowel disease patients who died: a retrospective study using the EICU-CRD database
Source: PLoS One. 2026 Jul 7;21(7):e0353237. doi: 10.1371/journal.pone.0353237 (PMC13340837; doi:10.1371/journal.pone.0353237)
Supplement: Attachment — Submitted filename: Response_to_Reviewers_auresp_2.docx [file pone.0353237.s003.docx]

Dear Reviewer,

Thank you for your careful review and valuable comments on our manuscript ”Association between hemoglobin-to-red cell distribution width ratio and occurrence of sepsis during ICU stay in inflammatory bowel disease patients who died: a retrospective study using the EICU-CRD database” (PONE-D-26-02302R1). We have made revisions according to your suggestions, as detailed below:

1、Regarding redundancy in the Results and Discussion

We have streamlined and reorganized the Results and Discussion sections to eliminate repetitive descriptions, thereby improving readability and avoiding redundancy.

2、Regarding predictive language such as "HRR holds potential clinical predictive value"

During the previous major revision, we had already carefully reviewed the full text and corrected all predictive statements. In this version, we re-examined every sentence to ensure all related descriptions now appropriately use phrasing such as "suggests an association" or "generates the hypothesis," consistent with the exploratory nature of this study. We apologize if you reviewed the pre-revision version. The issue has been fully addressed.

3、Regarding the "weighted quantile sum transformation" in statistical analysis

We agree with your comment that, given the sample size, standard logistic regression is sufficient. Therefore, we have removed the description of the weighted quantile sum transformation from the statistical analysis section.

4、Regarding the suggested additional references

We appreciate your recommendation. We have cited the three suggested studies in the Discussion section at appropriate points to enrich the discussion on composite biomarkers and non-linear relationships. The references are:

(11) doi:10.3389/fcimb.2025.1603104

(31) doi:10.3389/fendo.2025.1555082

(32)doi:10.3389/fcimb.2025.1602921

Once again, thank you for your time and professional guidance. We hope these revisions meet your expectations and bring the manuscript closer to acceptance. Please do not hesitate to let us know if further modifications are needed.

Sincerely,

The Authors
