# Supplementary material for: Association between hemoglobin-to-red cell distribution width ratio and occurrence of sepsis during ICU stay in inflammatory bowel disease patients who died: a retrospective study using the EICU-CRD database
Source: PLoS One. 2026 Jul 7;21(7):e0353237. doi: 10.1371/journal.pone.0353237 (PMC13340837; doi:10.1371/journal.pone.0353237)
Supplement: Peer review history

# Peer review: Decision Letter 0

**DOI:** 10.1371/journal.pone.0353237.r001

**Authors:** Samiullah Khan

14 Apr 2026

PONE-D-26-02302

Association between hemoglobin-to-red cell distribution width ratio and sepsis risk during ICU stay in inflammatory bowel disease patients who died: a retrospective study using the EICU-CRD database.

PLOS One

Dear Dr. li,

Thank you for submitting your manuscript to PLOS ONE. After careful consideration, we feel that it has merit but does not fully meet PLOS ONE’s publication criteria as it currently stands. Therefore, we invite you to submit a revised version of the manuscript that addresses the points raised during the review process.

Please submit your revised manuscript by May 28 2026 11:59PM. If you will need more time than this to complete your revisions, please reply to this message or contact the journal office at plosone@plos.org. When you're ready to submit your revision, log on to https://www.editorialmanager.com/pone/ and select the 'Submissions Needing Revision' folder to locate your manuscript file.

Please include the following items when submitting your revised manuscript:

- A letter that responds to each point raised by the academic editor and reviewer(s). You should upload this letter as a separate file labeled 'Response to Reviewers'.
- A marked-up copy of your manuscript that highlights changes made to the original version. You should upload this as a separate file labeled 'Revised Manuscript with Track Changes'.
- An unmarked version of your revised paper without tracked changes. You should upload this as a separate file labeled 'Manuscript'.

If you would like to make changes to your financial disclosure, please include your updated statement in your cover letter. Guidelines for resubmitting your figure files are available below the reviewer comments at the end of this letter.

If applicable, we recommend that you deposit your laboratory protocols in protocols.io to enhance the reproducibility of your results. Protocols.io assigns your protocol its own identifier (DOI) so that it can be cited independently in the future. For instructions see: https://journals.plos.org/plosone/s/submission-guidelines#loc-laboratory-protocols. Additionally, PLOS ONE offers an option for publishing peer-reviewed Lab Protocol articles, which describe protocols hosted on protocols.io. Read more information on sharing protocols at https://plos.org/protocols?utm_medium=editorial-email&utm_source=authorletters&utm_campaign=protocols.

As the corresponding author, your ORCID iD is verified in the submission system and will appear in the published article. PLOS supports the use of ORCID, and we encourage all coauthors to register for an ORCID iD and use it as well. Please encourage your coauthors to verify their ORCID iD within the submission system before final acceptance, as unverified ORCID iDs will not appear in the published article. *Only*  the individual author can complete the verification step; PLOS staff *cannot*  verify ORCID iDs on behalf of authors.

We look forward to receiving your revised manuscript.

Kind regards,

Samiullah Khan, Ph. D

Academic Editor

PLOS One

Journal Requirements:

When submitting your revision, we need you to address these additional requirements.

1.Please ensure that your manuscript meets PLOS ONE's style requirements, including those for file naming. The PLOS ONE style templates can be found at

https://journals.plos.org/plosone/s/file?id=wjVg/PLOSOne_formatting_sample_main_body.pdf and

https://journals.plos.org/plosone/s/file?id=ba62/PLOSOne_formatting_sample_title_authors_affiliations.pdf

2. Please note that PLOS One has specific guidelines on code sharing for submissions in which author-generated code underpins the findings in the manuscript. In these cases, we expect all author-generated code to be made available without restrictions upon publication of the work. Please review our guidelines at https://journals.plos.org/plosone/s/materials-and-software-sharing#loc-sharing-code and ensure that your code is shared in a way that follows best practice and facilitates reproducibility and reuse.

3. Thank you for stating the following financial disclosure:

“This work was supported by Reserve discipline construction of Jinshan Hospital Affiliated with Fudan University (No. ZDXK-2025-7)”

Please state what role the funders took in the study.  If the funders had no role, please state: "The funders had no role in study design, data collection and analysis, decision to publish, or preparation of the manuscript."

If this statement is not correct you must amend it as needed.

Please include this amended Role of Funder statement in your cover letter; we will change the online submission form on your behalf.

4. Please ensure that you refer to Figure 2 in your text as, if accepted, production will need this reference to link the reader to the figure.

5. Please include a separate caption for each figure in your manuscript.

6. Please upload a copy of Figure 4, to which you refer in your text on page 12. If the figure is no longer to be included as part of the submission please remove all reference to it within the text.

7. If the reviewer comments include a recommendation to cite specific previously published works, please review and evaluate these publications to determine whether they are relevant and should be cited. There is no requirement to cite these works unless the editor has indicated otherwise.

Additional Editor Comments:

Dear author,

Revise the manuscript extensively in the light of reviewer's comments and submit the revised version.

Thanks

[Note: HTML markup is below. Please do not edit.]

Reviewers' comments:

Reviewer's Responses to Questions

**Comments to the Author**

1. Is the manuscript technically sound, and do the data support the conclusions?

The manuscript must describe a technically sound piece of scientific research with data that supports the conclusions. Experiments must have been conducted rigorously, with appropriate controls, replication, and sample sizes. The conclusions must be drawn appropriately based on the data presented.

Reviewer #1: Yes

**********

2. Has the statistical analysis been performed appropriately and rigorously?

Reviewer #1: No

**********

3. Have the authors made all data underlying the findings in their manuscript fully available?

The PLOS Data policy requires authors to make all data underlying the findings described in their manuscript fully available without restriction, with rare exception (please refer to the Data Availability Statement in the manuscript PDF file). The data should be provided as part of the manuscript or its supporting information, or deposited to a public repository. For example, in addition to summary statistics, the data points behind means, medians and variance measures should be available. If there are restrictions on publicly sharing data—e.g. participant privacy or use of data from a third party—those must be specified.

Reviewer #1: Yes

**********

4. Is the manuscript presented in an intelligible fashion and written in standard English?

PLOS ONE does not copyedit accepted manuscripts, so the language in submitted articles must be clear, correct, and unambiguous. Any typographical or grammatical errors should be corrected at revision, so please note any specific errors here.

Reviewer #1: Yes

**********

5. Review Comments to the Author

Please use the space provided to explain your answers to the questions above. You may also include additional comments for the author, including concerns about dual publication, research ethics, or publication ethics. (Please upload your review as an attachment if it exceeds 20,000 characters)

Reviewer #1:

1. This study compared the relationship between sepsis and HRR in IBD patients who died in the ICU. The summary should emphasize that this is an analysis of the deceased population. Since the study does not involve predictive modeling, conclusions must be more rigorously stated.

2. The objectives and importance of the study are inadequate. Why only deceased patients are selected? There is a lack of theoretical basis and discussion about the mechanism of death in this disease.

3. In the methods part, "survival more than 30 days after ICU admission" as an exclusion criterion may introduce selection bias.

4. The sample size in statistical analysis was small, and there was a high risk of overfitting. Multicollinearity is not reported, and red blood cell counts are likely collinear with HRR.

5. The conclusion lacks rigor and contains overgeneralizations, and needs to be revised. The limitation should first address the selection bias that arises from the inclusion of only deceased patients.

**********

6. PLOS authors have the option to publish the peer review history of their article (what does this mean?). If published, this will include your full peer review and any attached files.

If you choose “no”, your identity will remain anonymous but your review may still be made public.

**Do you want your identity to be public for this peer review?**  For information about this choice, including consent withdrawal, please see our Privacy Policy.

Reviewer #1: **Yes:**  Zeyang Dong

**********

[NOTE: If reviewer comments were submitted as an attachment file, they will be attached to this email and accessible via the submission site. Please log into your account, locate the manuscript record, and check for the action link "View Attachments". If this link does not appear, there are no attachment files.]

To ensure your figures meet our technical requirements, please review our figure guidelines: https://journals.plos.org/plosone/s/figures

You may also use PLOS’s free figure tool, NAAS, to help you prepare publication quality figures: https://journals.plos.org/plosone/s/figures#loc-tools-for-figure-preparation.

NAAS will assess whether your figures meet our technical requirements by comparing each figure against our figure specifications.

# Author comment: Author response to Decision Letter 1

**DOI:** 10.1371/journal.pone.0353237.r002

21 May 2026

Dear Editor,

Thank you for your email and the opportunity to revise our manuscript. We have carefully addressed all the points raised by the reviewers in the previous round and ensured that the manuscript now fully complies with PLOS ONE’s style and formatting requirements as outlined in your email. Below, we provide a detailed, point-by-point response.

In addition to the academic revisions, we would like to respectfully bring one administrative circumstance to your attention. The first author of this study is currently completing standardized specialist training in Shanghai, with a graduation requirement of having a manuscript published or officially accepted by May 30, 2026. This timeline is crucial for their professional certification. We understand that the review process requires thoroughness and scientific rigor. However, given this pressing deadline, we would be most grateful if you and the reviewers could consider providing your decision and feedback as expeditiously as possible, to allow us the opportunity to meet this essential career requirement.

We assure you that this personal circumstance in no way diminishes our commitment to the highest standards of scientific integrity and transparency, which we have strived to uphold in this revised manuscript. We sincerely hope that our thorough revisions and this explanation will merit your positive consideration.

1.Style Requirements: We have thoroughly reviewed and reformatted our manuscript to adhere to PLOS ONE’s style templates. We confirm that the manuscript now meets all formatting requirements regarding file naming, document structure, and reference style.

2. Code Sharing: We acknowledge PLOS ONE’s policy on code sharing. Our study is a retrospective analysis based on the eICU-CRD database, and the primary statistical analyses were performed using R 4.4.3. The analysis does not rely on novel, author-generated computational code or algorithms that underpin the findings. The statistical methods employed (e.g., multivariate logistic regression, restricted cubic splines) are standard and implemented using common R packages.

3. Financial Disclosure: As stated in the Funding section of our manuscript, “This work was supported by Reserve discipline construction of Jinshan Hospital Affiliated with Fudan University (No. ZDXK-2025-7)” 21. We confirm that the funders had no role in study design, data collection and analysis, decision to publish, or preparation of the manuscript. Please amend the online submission form accordingly.

4. & 5. Figure References and Captions: We have reviewed the entire manuscript and confirm that all figures (including Figure 2) are appropriately referenced in the text. Each figure is now accompanied by a separate, detailed caption.

6. Figure 4: We apologize for the oversight. Reference to “Figure 4” on page 12 was an error from a previous draft. We have removed all mention of Figure 4 from the manuscript text. The current submission contains only the figures that are essential and referenced.

7. Reviewer-Recommended Citations: We have reviewed the publications suggested by the reviewers in their comments. We have evaluated their relevance to our study’s context—specifically, the association between HRR and outcomes in critical illness and sepsis. We have accordingly cited several key and relevant publications that support the biological rationale and clinical context of our findings in the revised Discussion section.

Sincerely,

The Authors

Dear Reviewer,

We are deeply grateful for your insightful and constructive comments, which have significantly improved the quality and clarity of our manuscript. We have carefully considered each point and made substantial revisions accordingly. Below, we provide a detailed, point-by-point response.

Reviewer # 1:

Comment 1: “This study compared the relationship between sepsis and HRR in IBD patients who died in the ICU. The summary should emphasize that this is an analysis of the deceased population. Since the study does not involve predictive modeling, conclusions must be more rigorously stated.”

Response:

We sincerely thank the reviewer for this crucial point.

We have thoroughly revised the manuscript to consistently emphasize the exclusive focus on the deceased patient subgroup and to ensure conclusions are descriptive and non-predictive.

In the Abstract, we now explicitly state the study population as “IBD patients who all died within 30 days of ICU admission” and describe it as an “extreme-prognosis subgroup”. The objective is framed as exploring “the association between admission HRR and the occurrence of sepsis during ICU stay in this specific subgroup”.

Throughout the Results and Discussion, we consistently refer to “this subgroup” and describe findings as “associations within this subgroup”.

The Conclusion has been strengthened to state with precision: “As all subjects were predetermined to be deceased patients, the findings can only describe associations within this subgroup and cannot be generalized to all IBD patients in ICU” . We have removed any language that could be misconstrued as predictive.

Comment 2: “The objectives and importance of the study are inadequate. Why only deceased patients selected? There is a lack of theoretical basis and discussion about the mechanism of death in this disease.”

Response:

We appreciate this request for greater clarity on the study’s rationale and mechanistic context.

Study Rationale: We have expanded the Introduction to better explain the selection of this unique cohort. We clarify that due to the specific constraints of the eICU-CRD database and our analytical focus, the study is inherently limited to analyzing IBD patients with the extreme outcome of death within 30 days 0. We acknowledge this introduces significant selection bias but argue that characterizing this “extreme-prognosis subgroup” has exploratory value for understanding the full spectrum of critical IBD .

Pathophysiological Discussion: In the Discussion, we have enriched the interpretation of our findings. We elaborate on how a low HRR in this non-survivor cohort likely reflects a compounded state of severe inflammation, anemia of chronic disease, and potential nutritional impairment, which may contribute to vulnerability to sepsis through mechanisms like gut barrier dysfunction and immune dysregulation, as suggested by related literature on systemic inflammation 11. This provides a stronger theoretical basis for the observed association in this critically ill population.

Comment 3: “In the methods part, ‘survival more than 30 days after ICU admission’ as an exclusion criterion may introduce selection bias.”

Response:

The reviewer is absolutely correct. This is not merely a potential bias but the defining characteristic and primary limitation of our study design. We have made this transparent and central to the manuscript.

We have reframed the Methods (Study Population) section to clearly state that the study population consists specifically of “IBD patients who died within 30 days of ICU admission” .

This selection bias is now the lead point in the Limitations section: “The most significant [limitation] is inherent selection bias. As we focused only on deceased patients, we lack insights into the prognostic value of HRR for sepsis among ICU-admitted IBD survivors” .

Comment 4: “The sample size in statistical analysis was small, and there was a high risk of overfitting. Multicollinearity is not reported, and red blood cell counts are likely collinear with HRR.”

Response: We thank the reviewer for these important methodological points.

Sample Size and Overfitting: We acknowledge the limited sample size. To mitigate overfitting in our multivariate models, we employed a parsimonious strategy, adjusting only for a limited set of key covariates (e.g., age, gender, race, weight, calcium, WBC, temperature) based on clinical relevance and univariate analysis 711. We have added a cautionary note in the Limitations section regarding this .

Multicollinearity: Following the reviewer’s suggestion, we formally assessed multicollinearity for the final adjusted model (Model 3). We calculated the Variance Inflation Factor (VIF) for all included variables. As shown in the supplementary material (Table S1), all VIF values were well below 10 (e.g., RBC count VIF = 5.41, HRR VIF = 2.01), indicating that severe multicollinearity was not present 13. We have added a statement in the Statistical Analysis section noting that multicollinearity was assessed and was not a concern.

Comment 5: “The conclusion lacks rigor and contains overgeneralizations, and needs to be revised. The limitation should first address the selection bias that arises from the inclusion of only deceased patients.”

Response: We agree entirely and have comprehensively revised the conclusion and limitations to ensure rigor and specificity.

Primary Limitation: As noted in response to Comment, the Limitations section now begins by directly addressing the selection bias .

Revised Conclusion: The Conclusion has been rewritten to be strictly accurate and limited: “Our analysis found that a lower HRR at admission was independently associated with an increased risk of sepsis during the ICU stay in this subgroup of deceased IBD patients. However, due to data limitations, there is significant selection bias in this study. As all subjects were predetermined to be deceased patients, the findings can only describe associations within this subgroup and cannot be generalized to all IBD patients in ICU” .

We believe these revisions have significantly strengthened the manuscript by enhancing its transparency, methodological rigor, and by precisely framing the scope and implications of the findings. We are grateful for the reviewer’s valuable input.

We sincerely thank you again for your time and constructive guidance, which has greatly improved the quality of our manuscript. We look forward to your further feedback.

Sincerely,

The Authors

Attachment Submitted filename: Response to Reviewers.docx

# Peer review: Decision Letter 1

**DOI:** 10.1371/journal.pone.0353237.r003

**Authors:** Samiullah Khan, Samiullah Khan

11 Jun 2026

PONE-D-26-02302R1

Association between hemoglobin-to-red cell distribution width ratio and occurrence of sepsis during ICU stay in inflammatory bowel disease patients who died: a retrospective study using the EICU-CRD database

PLOS One

Dear Dr. li,

Thank you for submitting your manuscript to PLOS ONE. After careful consideration, we feel that it has merit but does not fully meet PLOS ONE’s publication criteria as it currently stands. Therefore, we invite you to submit a revised version of the manuscript that addresses the points raised during the review process.

Please submit your revised manuscript by Jul 26 2026 11:59PM. If you will need more time than this to complete your revisions, please reply to this message or contact the journal office at plosone@plos.org. When you're ready to submit your revision, log on to https://www.editorialmanager.com/pone/ and select the 'Submissions Needing Revision' folder to locate your manuscript file.

Please include the following items when submitting your revised manuscript:

- A letter that responds to each point raised by the academic editor and reviewer(s). You should upload this letter as a separate file labeled 'Response to Reviewers'.
- A marked-up copy of your manuscript that highlights changes made to the original version. You should upload this as a separate file labeled 'Revised Manuscript with Track Changes'.
- An unmarked version of your revised paper without tracked changes. You should upload this as a separate file labeled 'Manuscript'.

If you would like to make changes to your financial disclosure, please include your updated statement in your cover letter. Guidelines for resubmitting your figure files are available below the reviewer comments at the end of this letter.

If applicable, we recommend that you deposit your laboratory protocols in protocols.io to enhance the reproducibility of your results. Protocols.io assigns your protocol its own identifier (DOI) so that it can be cited independently in the future. For instructions see: https://journals.plos.org/plosone/s/submission-guidelines#loc-laboratory-protocols. Additionally, PLOS ONE offers an option for publishing peer-reviewed Lab Protocol articles, which describe protocols hosted on protocols.io. Read more information on sharing protocols at https://plos.org/protocols?utm_medium=editorial-email&utm_source=authorletters&utm_campaign=protocols.

As the corresponding author, your ORCID iD is verified in the submission system and will appear in the published article. PLOS supports the use of ORCID, and we encourage all coauthors to register for an ORCID iD and use it as well. Please encourage your coauthors to verify their ORCID iD within the submission system before final acceptance, as unverified ORCID iDs will not appear in the published article. *Only*  the individual author can complete the verification step; PLOS staff *cannot*  verify ORCID iDs on behalf of authors.

We look forward to receiving your revised manuscript.

Kind regards,

Samiullah Khan, Ph. D

Academic Editor

PLOS One

Journal Requirements:

1. If the reviewer comments include a recommendation to cite specific previously published works, please review and evaluate these publications to determine whether they are relevant and should be cited. There is no requirement to cite these works unless the editor has indicated otherwise.

2. Please review your reference list to ensure that it is complete and correct. If you have cited papers that have been retracted, please include the rationale for doing so in the manuscript text, or remove these references and replace them with relevant current references. Any changes to the reference list should be mentioned in the rebuttal letter that accompanies your revised manuscript. If you need to cite a retracted article, indicate the article’s retracted status in the References list and also include a citation and full reference for the retraction notice.

**Additional Editor Comments:**

Dear author,

Address all the queries raised by reviewer 2 and submit the revised version accordingly.

[Note: HTML markup is below. Please do not edit.]

Reviewers' comments:

Reviewer's Responses to Questions

**Comments to the Author**

1. If the authors have adequately addressed your comments raised in a previous round of review and you feel that this manuscript is now acceptable for publication, you may indicate that here to bypass the “Comments to the Author” section, enter your conflict of interest statement in the “Confidential to Editor” section, and submit your "Accept" recommendation.

Reviewer #1: All comments have been addressed

Reviewer #2: All comments have been addressed

**********

2. Is the manuscript technically sound, and do the data support the conclusions?

The manuscript must describe a technically sound piece of scientific research with data that supports the conclusions. Experiments must have been conducted rigorously, with appropriate controls, replication, and sample sizes. The conclusions must be drawn appropriately based on the data presented.

Reviewer #1: Yes

Reviewer #2: Yes

**********

3. Has the statistical analysis been performed appropriately and rigorously?

Reviewer #1: Yes

Reviewer #2: Yes

**********

4. Have the authors made all data underlying the findings in their manuscript fully available?

The PLOS Data policy requires authors to make all data underlying the findings described in their manuscript fully available without restriction, with rare exception (please refer to the Data Availability Statement in the manuscript PDF file). The data should be provided as part of the manuscript or its supporting information, or deposited to a public repository. For example, in addition to summary statistics, the data points behind means, medians and variance measures should be available. If there are restrictions on publicly sharing data—e.g. participant privacy or use of data from a third party—those must be specified.

Reviewer #1: Yes

Reviewer #2: Yes

**********

5. Is the manuscript presented in an intelligible fashion and written in standard English?

PLOS ONE does not copyedit accepted manuscripts, so the language in submitted articles must be clear, correct, and unambiguous. Any typographical or grammatical errors should be corrected at revision, so please note any specific errors here.

Reviewer #1: Yes

Reviewer #2: Yes

**********

6. Review Comments to the Author

Please use the space provided to explain your answers to the questions above. You may also include additional comments for the author, including concerns about dual publication, research ethics, or publication ethics. (Please upload your review as an attachment if it exceeds 20,000 characters)

Reviewer #1: The author has largely addressed the issues previously raised, the paper may be considered for acceptance.

Reviewer #2: The authors have addressed most of the previous concerns commendably. They have clearly emphasized the selection bias, reframed the objective as descriptive, and improved the transparency of the limitations. The study now appropriately presents itself as an exploratory analysis of a deceased-only subgroup. However, a few minor issues remain before the manuscript can be accepted.

First, the manuscript contains substantial repetition, particularly in the Results and Discussion sections. For example, the description of the association between HRR and sepsis (OR, quartile analysis, RCS) is repeated almost verbatim in consecutive paragraphs. The authors should streamline these sections to improve readability and avoid redundancy.

Second, some statements still lean towards predictive language despite the revisions. For instance, the last sentence of the abstract (page 1) and the conclusion (page 65) state “HRR holds potential clinical predictive value”. Since the study is purely descriptive in a selected deceased cohort, such wording should be revised to “suggests an association” or “generates the hypothesis that HRR may be associated with sepsis risk in this specific subgroup”. Please adjust similar phrasing throughout.

Third, the authors mention “weighted quantile sum transformation for continuous covariates” in the statistical analysis but do not explain why this was applied or how it was implemented. Given the sample size, a standard logistic regression without this transformation would be sufficient. The authors should either justify its use or remove it.

Fourth, to strengthen the methodological context and demonstrate awareness of recent literature on composite biomarkers in critical illness, the authors are encouraged to cite the following relevant studies in the Discussion (e.g., when discussing the role of ratio-based markers or dose-response relationships): (1) a study on the J‑shaped association between neutrophil‑to‑PNI ratio and mortality in sepsis (doi:10.3389/fcimb.2025.1603104); (2) a study on the creatinine‑to‑albumin ratio as a prognostic marker in sepsis‑associated AKI (doi:10.3389/fcimb.2025.1602921); and (3) a study on the glucose‑potassium ratio for short‑ and long‑term mortality prediction in sepsis (doi:10.3389/fendo.2025.1555082). These references are not required for acceptance but would enrich the discussion of non‑linear relationships and the utility of composite biomarkers.

After these minor revisions, the manuscript will be suitable for publication. I recommend minor revision.

**********

7. PLOS authors have the option to publish the peer review history of their article (what does this mean?). If published, this will include your full peer review and any attached files.

If you choose “no”, your identity will remain anonymous but your review may still be made public.

**Do you want your identity to be public for this peer review?**  For information about this choice, including consent withdrawal, please see our Privacy Policy.

Reviewer #1: No

Reviewer #2: **Yes:**  Jiaqi Lou

**********

[NOTE: If reviewer comments were submitted as an attachment file, they will be attached to this email and accessible via the submission site. Please log into your account, locate the manuscript record, and check for the action link "View Attachments". If this link does not appear, there are no attachment files.]

To ensure your figures meet our technical requirements, please review our figure guidelines: https://journals.plos.org/plosone/s/figures

You may also use PLOS’s free figure tool, NAAS, to help you prepare publication quality figures: https://journals.plos.org/plosone/s/figures#loc-tools-for-figure-preparation.

NAAS will assess whether your figures meet our technical requirements by comparing each figure against our figure specifications.

# Author comment: Author response to Decision Letter 2

**DOI:** 10.1371/journal.pone.0353237.r004

15 Jun 2026

Dear Reviewer,

Thank you for your careful review and valuable comments on our manuscript ”Association between hemoglobin-to-red cell distribution width ratio and occurrence of sepsis during ICU stay in inflammatory bowel disease patients who died: a retrospective study using the EICU-CRD database” (PONE-D-26-02302R1). We have made revisions according to your suggestions, as detailed below:

1、Regarding redundancy in the Results and Discussion

We have streamlined and reorganized the Results and Discussion sections to eliminate repetitive descriptions, thereby improving readability and avoiding redundancy.

2、Regarding predictive language such as "HRR holds potential clinical predictive value"

During the previous major revision, we had already carefully reviewed the full text and corrected all predictive statements. In this version, we re-examined every sentence to ensure all related descriptions now appropriately use phrasing such as "suggests an association" or "generates the hypothesis," consistent with the exploratory nature of this study. We apologize if you reviewed the pre-revision version. The issue has been fully addressed.

3、Regarding the "weighted quantile sum transformation" in statistical analysis

We agree with your comment that, given the sample size, standard logistic regression is sufficient. Therefore, we have removed the description of the weighted quantile sum transformation from the statistical analysis section.

4、Regarding the suggested additional references

We appreciate your recommendation. We have cited the three suggested studies in the Discussion section at appropriate points to enrich the discussion on composite biomarkers and non-linear relationships. The references are:

(11) doi:10.3389/fcimb.2025.1603104

(31) doi:10.3389/fendo.2025.1555082

(32)doi:10.3389/fcimb.2025.1602921

Once again, thank you for your time and professional guidance. We hope these revisions meet your expectations and bring the manuscript closer to acceptance. Please do not hesitate to let us know if further modifications are needed.

Sincerely,

The Authors

Attachment Submitted filename: Response_to_Reviewers_auresp_2.docx

# Peer review: Decision Letter 2

**DOI:** 10.1371/journal.pone.0353237.r005

**Authors:** Samiullah Khan, Samiullah Khan, Samiullah Khan

21 Jun 2026

Association between hemoglobin-to-red cell distribution width ratio and occurrence of sepsis during ICU stay in inflammatory bowel disease patients who died: a retrospective study using the EICU-CRD database

PONE-D-26-02302R2

Dear Dr. Yulin Li,

We’re pleased to inform you that your manuscript has been judged scientifically suitable for publication and will be formally accepted for publication once it meets all outstanding technical requirements.

Within one week, you’ll receive an e-mail detailing the required amendments. When these have been addressed, you’ll receive a formal acceptance letter and your manuscript will be scheduled for publication.

An invoice will be generated when your article is formally accepted. Please note, if your institution has a publishing partnership with PLOS and your article meets the relevant criteria, all or part of your publication costs will be covered. Please make sure your user information is up-to-date by logging into Editorial Manager at Editorial Manager® and clicking the ‘Update My Information' link at the top of the page. For questions related to billing, please contact billing support.

If your institution or institutions have a press office, please notify them about your upcoming paper to help maximize its impact. If they’ll be preparing press materials, please inform our press team as soon as possible -- no later than 48 hours after receiving the formal acceptance. Your manuscript will remain under strict press embargo until 2 pm Eastern Time on the date of publication. For more information, please contact onepress@plos.org.

Kind regards,

Samiullah Khan, Ph. D

Academic Editor

PLOS One

Additional Editor Comments (optional):

Dear author,

You have addressed all the queries raised by both reviewers, both reviewers are satisfied. Therefore suggest that manuscript is now acceptable for publication in PLOS ONE.

Reviewers' comments:

Reviewer's Responses to Questions

**Comments to the Author**

1. If the authors have adequately addressed your comments raised in a previous round of review and you feel that this manuscript is now acceptable for publication, you may indicate that here to bypass the “Comments to the Author” section, enter your conflict of interest statement in the “Confidential to Editor” section, and submit your "Accept" recommendation.

Reviewer #2: All comments have been addressed

**********

2. Is the manuscript technically sound, and do the data support the conclusions?

The manuscript must describe a technically sound piece of scientific research with data that supports the conclusions. Experiments must have been conducted rigorously, with appropriate controls, replication, and sample sizes. The conclusions must be drawn appropriately based on the data presented.

Reviewer #2: Yes

**********

3. Has the statistical analysis been performed appropriately and rigorously?

Reviewer #2: Yes

**********

4. Have the authors made all data underlying the findings in their manuscript fully available?

The PLOS Data policy requires authors to make all data underlying the findings described in their manuscript fully available without restriction, with rare exception (please refer to the Data Availability Statement in the manuscript PDF file). The data should be provided as part of the manuscript or its supporting information, or deposited to a public repository. For example, in addition to summary statistics, the data points behind means, medians and variance measures should be available. If there are restrictions on publicly sharing data—e.g. participant privacy or use of data from a third party—those must be specified.

Reviewer #2: Yes

**********

5. Is the manuscript presented in an intelligible fashion and written in standard English?

PLOS ONE does not copyedit accepted manuscripts, so the language in submitted articles must be clear, correct, and unambiguous. Any typographical or grammatical errors should be corrected at revision, so please note any specific errors here.

Reviewer #2: Yes

**********

6. Review Comments to the Author

Please use the space provided to explain your answers to the questions above. You may also include additional comments for the author, including concerns about dual publication, research ethics, or publication ethics. (Please upload your review as an attachment if it exceeds 20,000 characters)

Reviewer #2: I have re-reviewed the revised version of this manuscript (R2). The authors have addressed all concerns raised in the previous round of review in a thorough and satisfactory manner.

Minor remaining issues (not requiring further review):

In Table 3, the VIF for HGB (5.83), RBC (5.41), and RDW (1.61) are all below 10, indicating no severe multicollinearity. However, since HRR is derived from Hb and RDW, including all three in the same model may still introduce collinearity concerns. The authors might consider clarifying why these variables were all included despite the derivation.

In the Discussion (lines 238-240 on page 47), there is a duplicated sentence fragment ("We found that a lower HRR was strongly and independently associated...") that appears immediately after the paragraph summarizing the findings. This should be removed.

The abstract states "each 1-unit increase in HRR was associated with 20.3% lower odds of sepsis (OR = 0.797, 95% CI: 0.672-0.937; P = 0.007)." In the main text (Table 2, Model 3), the corresponding OR is 0.779. Please ensure consistency between abstract and main text.

These are minor editorial issues that can be corrected during proofreading and do not require further peer review.

The authors have produced a well-structured, honestly reported, and methodologically sound exploratory study. The manuscript is appropriately cautious in its conclusions and transparent about its limitations. The findings generate a testable hypothesis regarding HRR as a potential biomarker in end-stage IBD that merits validation in prospective cohorts.

**********

7. PLOS authors have the option to publish the peer review history of their article (what does this mean?). If published, this will include your full peer review and any attached files.

If you choose “no”, your identity will remain anonymous but your review may still be made public.

**Do you want your identity to be public for this peer review?**  For information about this choice, including consent withdrawal, please see our Privacy Policy.

Reviewer #2: **Yes:**  Jiaqi Lou

**********

# Editor report: Acceptance letter

**DOI:** 10.1371/journal.pone.0353237.r006

**Authors:** Samiullah Khan, Samiullah Khan, Samiullah Khan

PONE-D-26-02302R2

PLOS One

Dear Dr. li,

I'm pleased to inform you that your manuscript has been deemed suitable for publication in PLOS One. Congratulations! Your manuscript is now being handed over to our production team.

At this stage, our production department will prepare your paper for publication. This includes ensuring the following:

* All references, tables, and figures are properly cited

* All relevant supporting information is included in the manuscript submission,

* There are no issues that prevent the paper from being properly typeset

You will receive further instructions from the production team, including instructions on how to review your proof when it is ready. Please keep in mind that we are working through a large volume of accepted articles, so please give us a few days to review your paper and let you know the next and final steps.

Lastly, if your institution or institutions have a press office, please let them know about your upcoming paper now to help maximize its impact. If they'll be preparing press materials, please inform our press team within the next 48 hours. Your manuscript will remain under strict press embargo until 2 pm Eastern Time on the date of publication. For more information, please contact onepress@plos.org.

You will receive an invoice from PLOS for your publication fee after your manuscript has reached the completed accept phase. If you receive an email requesting payment before acceptance or for any other service, this may be a phishing scheme. Learn how to identify phishing emails and protect your accounts at https://explore.plos.org/phishing.

If we can help with anything else, please email us at customercare@plos.org.

Thank you for submitting your work to PLOS ONE and supporting open access.

Kind regards,

PLOS ONE Editorial Office Staff

on behalf of

Dr. Samiullah Khan

Academic Editor

PLOS One
